# IR GRIN lenses prepared by ionic exchange in chalcohalide glasses

**DOI:** 10.1038/s41598-021-90626-4

**Published:** 2021-05-26

**Authors:** Claire Fourmentin, Xiang-Hua Zhang, Enora Lavanant, Thierry Pain, Mathieu Rozé, Yann Guimond, Francis Gouttefangeas, Laurent Calvez

**Affiliations:** 1 ISCR (Institut Des Sciences Chimiques de Rennes) - UMR 6226, CNRS, Univ Rennes, 35000 Rennes, France; 2 Umicore I.R Glass, Z.A du Boulais, 35690 Acigné, France; 3 ScanMAT-CMEBA-UMS2001, CNRS, Univ Rennes, 35000 Rennes, France

**Keywords:** Glasses, Materials for devices, Materials for optics

## Abstract

In order to decrease the number of lenses and the weight of thermal imaging devices, specific optical design are required by using gradient refractive index (GRIN) elements transparent in the infrared waveband. While widely used for making visible GRIN lenses with silicate glasses, the ion exchange process is very limited when applied to chalcogenide glasses due to their low T_g_ and relatively weak mechanical properties. In this paper, we develop chalco-halide glasses based on alkali halide (NaI) addition in a highly covalent GeSe_2_–Ga_2_Se_3_ matrix, efficient for tailoring a significant and permanent change of refractive by ion exchange process between K^+^ and Na^+^. Optical and structural properties of the glass samples were measured showing a diffusion length reaching more than 2 mm and a Gaussian gradient of refractive index Δn of 4.5.10^–2^. The obtained GRIN lenses maintain an excellent transmission in the second (3–5 µm) and third (8–12 µm) atmospheric windows.

## Introduction

To perform lightweight and compact IR systems for thermal imaging applications working in the third atmospheric window (8–12µm), this paper focuses on the development of radial IR GRIN lenses based on chalcogenide glasses. Recently, IR GRIN lenses were developed by applying a gradient of heat treatment to create axial^1^ and radial^2^ gradient of refractive index based on GeSe_2_–As_2_Se_3_–Pbse and GeSe_2_–Ga_2_Se_3_ systems, respectively. The key of success of this technology is the use of unstable chalcogenide glass composition against crystallization and a reproducible and fine control of crystallization is the key issue. However, the main drawback rely on the use of small quantity of glass that can be synthesized without crystallization. Our investigation focuses on an ionic exchange process on glasses of high stability against crystallization. Ion exchange process has proved to be highly successful with oxide-based glasses. The ion exchange process is a well-known technique used to modify the optical, mechanical or chemical properties on the glass surface, which will result in a multitude of advantages and applications such as the chemical strengthening of the surface of a glass^3–6^. In addition, the change of the composition of the glass surface allows the manufacture of planar waveguides^7,8^ as well as in our study, the manufacture of lenses with gradient of refractive index^9–12^.

Up to now, while highly developed in silicate glasses, the ion exchange process is still very little studied in chalcogenide glass family because of the lack of composition with mobile ions and their low glass transition temperature compared to that of silicates glasses wherein this process is widespread. Indeed, in conventional and commercial chalcogenide glasses derived from Ge-As-Se, As-Se or Ge-Sb-Se systems, the atoms are connected to each other by covalent bonds and there is no mobile ion to be exchanged.

To induce significant ionic mobility within the matrix, specific compositions based on chalco-halide were designed. The incorporation of alkali halide such as NaI within chalcogenide glassy matrix is already reported in several papers^13,14^. Wang et al.^15^ were the first to study ion exchange in sulfur-based glasses. Diffusion depths of K+ cations in a GeS_2_–Ga_2_S_3_–AgX system glass (with X = Cl, Br, I) greater than 250 μm were measured. However, the IR transmissions and other thermo-mechanical properties of the glasses were not presented. Later, this process has already been developed to enhance the mechanical properties of chalcogenide glasses based on selenium by Rozé et al.^16^. The compression of the glass surface is accomplished by replacing the K^+^ cations from the glass by Rb^+^ cations from the melt bath, which have a higher ionic radius (K^+^: 1.38 Å and Rb^+^: 1.52 Å). The insertion of bigger ions inside the restricted spaces on the surface will create compression forces. The glass is reinforced by the surfaces in compression and the heart in a state of compensatory tension. However, in this case, critical diffusion length of 25µm is reached before inducing strong deterioration of the base glass leading to irreversible damages due to inner mechanical constrains.

For making infrared lenses transparent in the second and third atmospheric windows, selenium-based glasses were selected. To maintain a glass transition temperature (T_g_) higher than 300°C because of the used molten baths, alkali halide was incorporated in high-T_g_ glasses belonging to the Ge-Ga-Se ternary system. Based on these glasses, the (72 GeSe_2_–28 Ga_2_Se_3_)_75_ (MI)_25_ glass equivalent to the Ge_17_Ga_13_Se_54_ I_8_M_8_ (at%.) composition was selected due to its high rate of alkali ions (M = Na^+^, K^+^ or a mix of both).

Another key parameter of the ion exchange process is the composition of the exchange bath and its resulting melting temperature. Indeed, the melting temperature of the bath must be lower than the glass transition temperature of the samples to avoid their deformation during the ion exchange. The choice of the bath composition was focused on the nitrate compounds because of their low vapor pressure when melted, relatively low melting temperatures, and high dissolution rate in water to remove it easily from glass surface.

## Results

### Diffusion in chalco-halide glasses

Thermal characteristics of the base glass containing 25%mol. of NaI are presented in the Table 1 and compared to the base glass without alkali halide.

**Table 1:** Main Physical and optical characteristics of the studied glasses.

	**72 GeSe**_**2**_**-28 Ga**_**2**_**Se**_**3**_	**(72 GeSe**_**2**_**-28 Ga**_**2**_**Se**_**3**_**)**_**75**_** – (NaI)**_**25**_
T_g_ (± 2 °C)	372	310
T_x_ (± 2 °C)	430	428
Tx-Tg	58	118
n @ 1311 µm	2,415	2,248
n @ 1551 µm	2,405	2,241
TEC (± 0.2.10^–6^ K^-1^)	11,7	16,7
Density (± 0.02 g.cm^-3^)	4,43	4,20
Hv (± 8)	192	143
E (± 2 GPa)	22	19

As expected, the glass Tg decreases with addition of sodium iodine while the crystallization temperature remains unchanged leading to a high increase of the glass stability against crystallization. Indeed, weaker bonds appear in the glass due to Na^+^ cations, the crosslinking of the vitreous network is lower and the T_g_ is thus reduced^14^. Considering the fact that the exchange bath presents a melting point above 237°C, adding 25% of NaI is still optimal to keep a large range of working temperature. Also, one can notice a strong increase of the thermal expansion coefficient and a decrease of refractive index when adding NaI.

**Figure 1:**
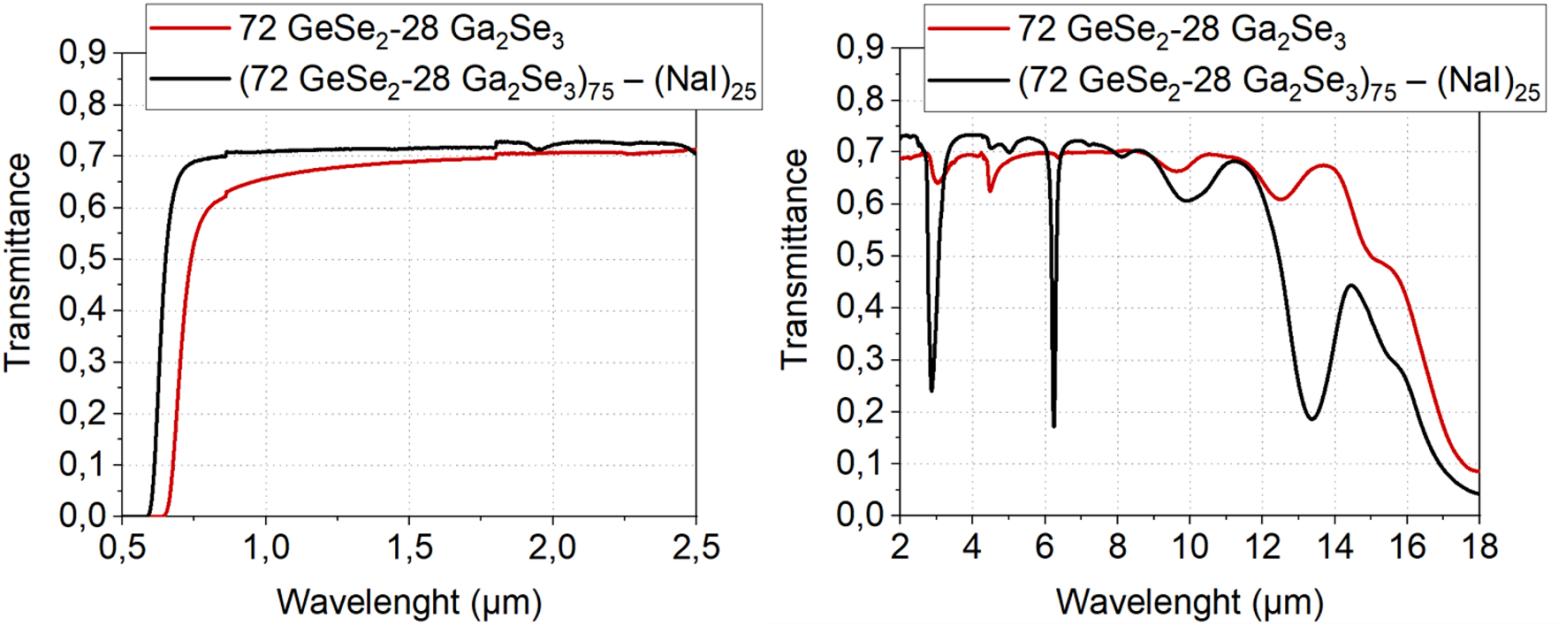
IR transmission of the 72 GeSe_2_–28 Ga_2_Se_3_ and the (72 GeSe_2_–28 Ga_2_Se_3_)_75_ (NaI)_25_ glasses.

Transmission windows of both glasses are presented in Figure 1. The addition of alkali halide induces a blue-shift of the beginning of transmission extending the transmission into the visible region but at the meantime introduces parasitic absorption bands due to O-H, Ge-O bonds. It is known that the introduction of alkali halide within covalent chalcogenide glassy matrix gives a higher hygroscopic behavior of the materials^14^.

**Figure 2:**
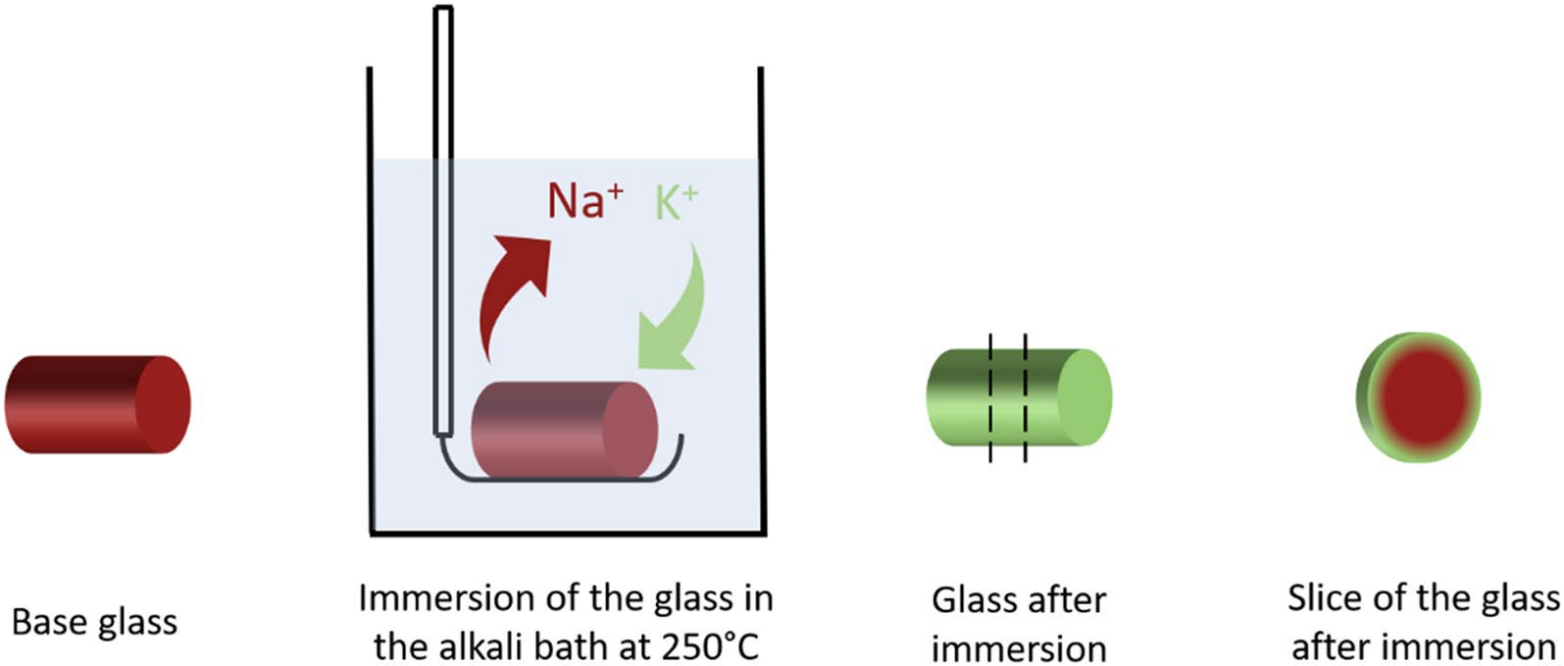
Experimental process of the ionic exchange and characterization.

Figure 2 presents a schema of the experimental process of ionic exchange.

**Figure 3:**
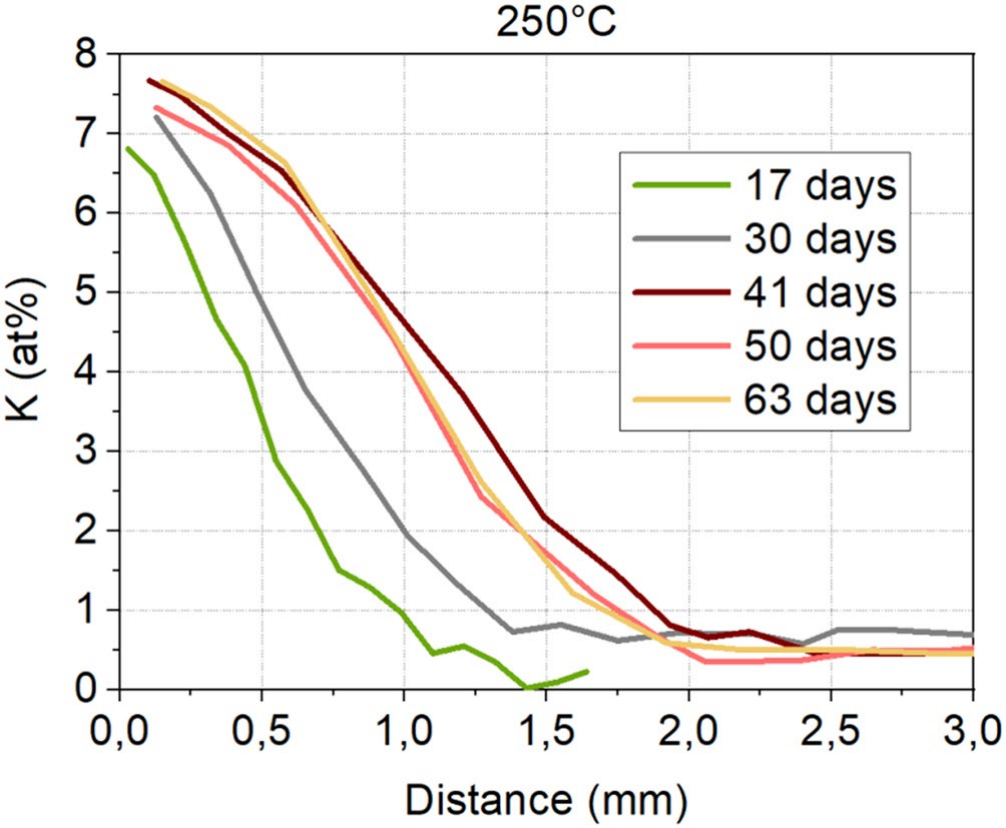
Potassium concentration profile in the (72 GeSe_2_–28 Ga_2_Se_3_)_75_ (NaI)_25_ glass after immersion in the 60 KNO_3_ / 40 NaNO_3_ bath for 1 h to 63 days.

As presented in Figure 2, polished samples of the (72 GeSe_2_–28 Ga_2_Se_3_)_75_ – (NaI)_25_ glass in rod shape of 10mm of diameter and 10mm thick were immersed in the 60 KNO_3_ / 40 NaNO_3_ bath (T_m_ of 237°C) at T_g_-60°C (250°C) for different durations, from 1h to 63 days. In order to focus on radial exchange, a slice of 4mm thick was cut in the middle of the 10 mm rod and the surfaces were polished then. The symmetrical nature of ion exchange was systematically checked by EDS on all the samples showing a perfect radial symmetry. The Fig. 3 presents only one side for a better read of curves.

The profile of potassium concentration was measured on the surface, from the edge to the center. As observable in Fig. 3, which presents the diffusion profile of K^+^ in the glass containing 25% of NaI immersed at 250°C, the potassium concentration profiles show a gradient of potassium diffusion into the glass. The diffusion depth increases with increasing time of immersion up to 2 mm after 40 days and is followed by a saturation step of diffusion. A rate of 8 at%. of potassium is reached at the edge of the samples, which means that all sodium from the based glass composition of Ge_17_Ga_13_Se_54_ I_8_Na_8_ (at%.) has been replaced by potassium ions. The new composition of the edge of the glass is then Ge_17_Ga_13_Se_54_ I_8_K_8_ (at%.) that is to say a glass composition of (72 GeSe_2_ – 28 Ga_2_Se_3_)_75_ (KI)_25_.

**Figure 4:**
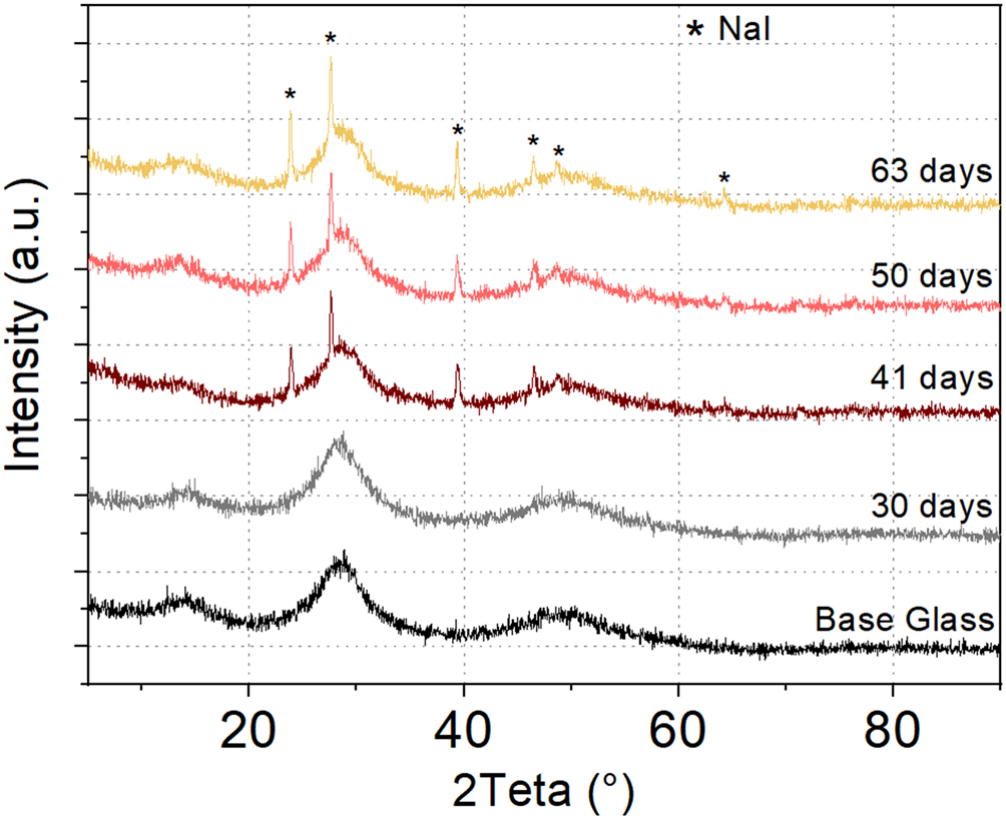
RX Diffractogram of the (72 GeSe_2_–28 Ga_2_Se_3_)_75_ (NaI)_25_ glass before immersion and after 30, 41, 50 and 63 days of immersion in the 60 KNO_3_ / 40 NaNO_3_ bath.

Figure 4 shows the RX diffractograms of the samples before and after being immersed from 30 to 63 days. The diffractogram was realized on bulk and powder to exclude potential surface crystallization.

After 40 days of immersion, a crystallization of sodium iodine appears. After crystallization, sodium ions are less mobile to be exchanged with potassium ions, which could be an explanation of the saturation step of diffusion that we observed in Fig. 3.

**Figure 5:**
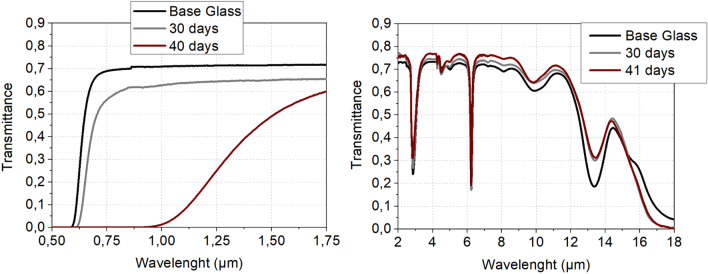
IR transmission of (72 GeSe2–28 Ga2Se3)75 (NaI)_25_ samples before and after immersion for different duration in the 60 KNO_3_/40 NaNO_3_ bath at 250 °C.

The transparency window of the immersed samples cut and polished are shown Fig. 5.

At short wavelength, a progressive shift of the band gap towards higher wavelength is observable for increased time of immersion. This result is consistent with Rayleigh scatterings due to submicron particles already observed by XRD. The maximum of transmittance is barely unchanged in the third atmospheric window between 8 and 14µm before and after the ion exchange. Moreover, no additional bonds due to oxidation of the glass are observed in this optical window dedicated for thermal imaging. This method is therefore entirely suitable for creating GRIN lenses made of chalcogenide glasses since the transmission of the glasses in the IR remains intact.

### Obtention of GRIN lenses

**Figure 6:**
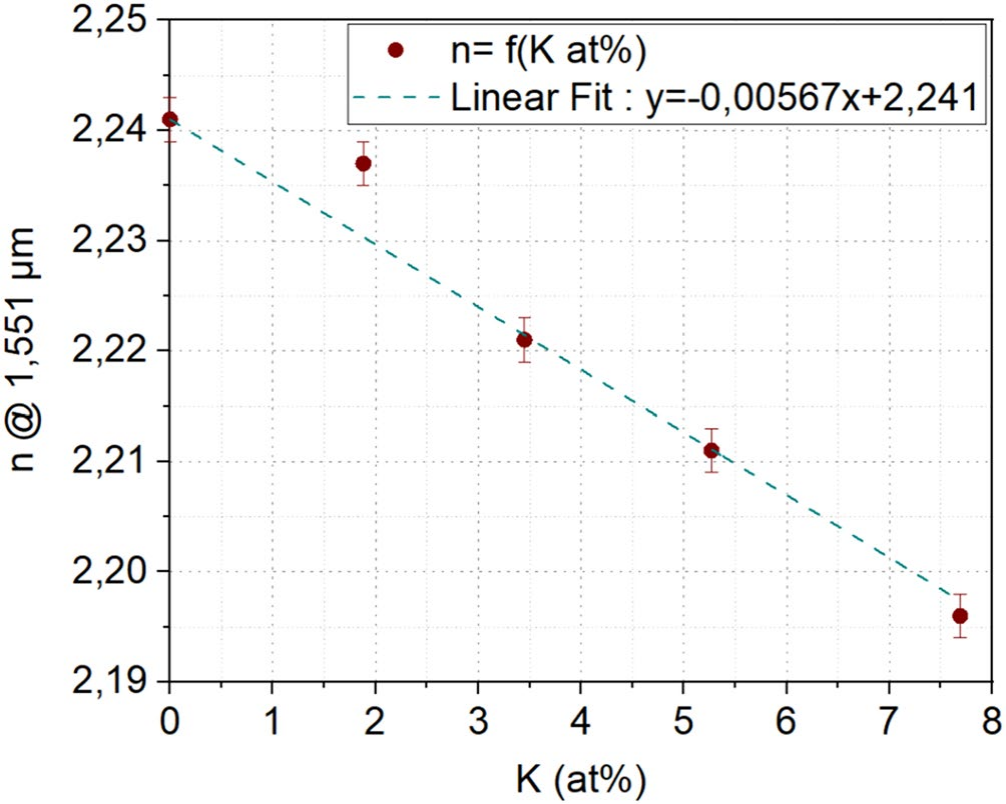
Glass index as a function of the rate of potassium ions in the (72 GeSe_2_–28 Ga_2_Se_3_)_75_ (KI_x_–NaI_1−x_)_25_ glass composition with x = 0, 0.25, 0.5, 0.75 and 1.

In order to determine the evolution of refractive index according to the proportion of alkaline exchanged, optical properties of glasses with a mix of sodium and potassium of different proportions were determined. The studied compositions are the following ones: (72 GeSe_2_–28 Ga_2_Se_3_)_75_ (KI_x_–NaI_1-x_)_25_ glasses with x = 0, 0.25, 0.5, 0.75 and 1. Figure 6 presents the evolution of the refractive index as a function of the atomic percentage of potassium in these glass compositions.

Those data show that the refractive index decreases linearly according to the potassium rate inside the glass. Therefore, a gradient of potassium in the glass leads to a gradient of index that is proportional to the potassium content, with a maximum ∆n reachable of -4,5.10^-2^ when all Na^+^ is replaced by K^+^ ions.

**Figure 7:**
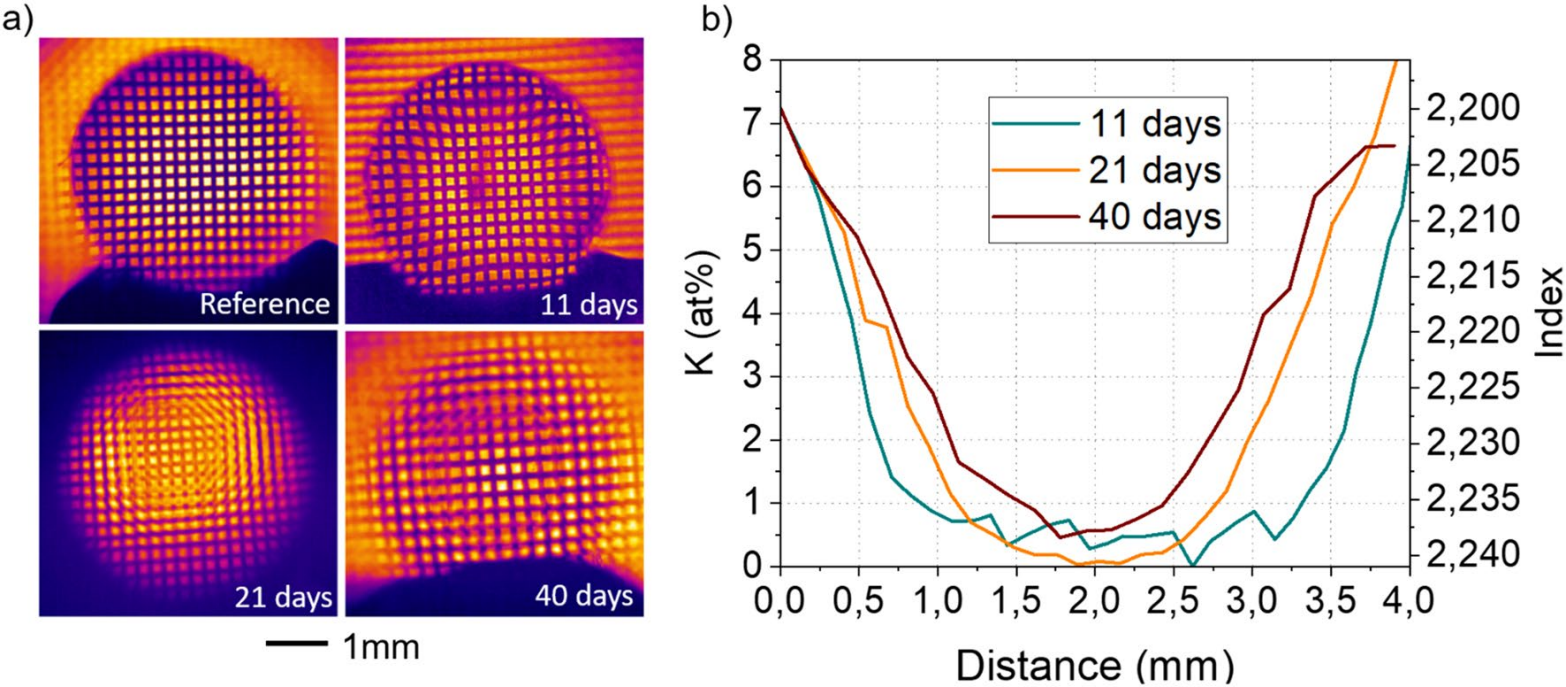
(**a**) GRIN lenses obtained by ionic exchange and (**b**) their respective potassium concentration and index profile.

In order to be in line with the maximum diffusion scale of 2mm of potassium in the glass, glass rods of 4 mm diameter were immersed from 11 days up to 40 days in the nitrate bath, in order to avoid crystallization. The GRIN lenses obtained as well as their characteristics are presented in Figure 7.

The observation of a metallic grid/lattice throughout the samples using a 8-12µm IR camera allows witnessing the variation of refraction index induced by ionic exchange **(**Figure 7a). As expected, the grid appears more and more curved as the diffusion depth increases.

The timescale allows for an easy control of the diffusion profile leading to a hyperbolic secant profile of refractive index with a maximum ∆n of −4.5×10^-2^ as shown in Figure7b). Beyond 40 days, an interdiffusion of K^+^ derived from both sides leads to a K^+^ concentration increase of at the center of the sample. This phenomenon can be the root cause of the slightly reduced Δn observed.

## Discussion

An efficient ionic exchange between Na^+^ and K^+^ has been realized by using optimized chalco-halide glass in molted mixed nitrates (NaNO_3_/KNO_3_) bath. The addition of amount of alkali halide (NaI) within a highly covalent matrix (Ge–Ga–Se) permits the creation of mobile ions needed for ionic exchange process while maintaining a high glass transition temperature. By controlling the time and temperature of experiment, diffusion depth of more than 2mm is reached without inducing any perturbations of the glass transmission in the second (3–5µm) and third (8–12µm) atmospheric windows. To our best knowledge, this is the first work reporting a so high diffusion depth induced in chalcogenide glasses combined to the generation of a permanent and intense (Δn=−4.5×10^-2^) change of refractive index. Our material is thus an ideal candidate for thermal imaging applications, in which compact embedded IR optics are needed.

## Methods

Chalcogenide glasses were prepared by following the ordinary melt/quenching method. All the raw elements (Ge, Ga, Se: 5N and NaI, KI: 2N) were weighted respectively to their composition. They were placed in a silica tube of 4 or 10 mm inner diameter, which was sealed under secondary vacuum (10^−5^ mbar). The mixture was heated up to 870°C for 10 hours and then quenched in water before being annealed for 3h at T_g_ to relax the mechanical constrains. In this preliminary experiment, no further steps of purification were performed. Glass rods of 10mm high were cut and then finely polished for optical characterization and ion exchange experiments.

The ion exchange process is focused on Na^+^/K^+^ because of their close ionic radius (Na^+^: 1.02 Å and K^+^: 1.38 Å). The melting temperature of KNO_3_ is 334°C, which prevents from using it alone for ionic exchange experiment without deteriorating the glass samples. Thus, a mix of two nitrates based on sodium and potassium was selected to decrease the melting temperature of the bath. The 60 KNO_3_ / 40 NaNO_3_ bath composition was selected because it presents a good compromise between a relatively low melting temperature (237°C) and a high content of potassium. The bath was prepared by melting and mixing the two compounds above their melting temperature, thus above 350°C, and then was cooled down at the working temperature.

The samples were immersed for different duration at 250°C in a silica chamber containing the melted alkali nitrate bath. This low temperature is optimal to offer a slow diffusion rate that allows a good control of the diffusion length without deteriorating the chalcogenide glass. After the ion exchange, the glass rods are then rinsed under distilled water to remove residual melt bath on the surface.

To obtain radial graded index, the samples having undergone ion exchange process were cut into a slice of 4mm thick in the middle of the 10mm length rod and the surfaces were finely polished, as depictured in Fig. 2. The chemical concentration profile of K^+^ was measured on the surface, from the edge to the center using a Scanning Electron Microscope (JEOL IT 300 LA) equipped with an Energy Dispersive X-Ray Spectroscopy (EDS).

Differential Scanning Calorimetry experiments (DSC 2010 TA Q20) were performed to measure the characteristic temperatures (glass transition temperature: T_g_, temperature of crystallization: T_x_) of the glass with a heating rate of 10 °C/min.

The transmission was measured using UV–vis and FTIR spectrophotometer. To obtain the refractive index as a function of the wavelength of the glass surface, the so-called m-line technique was used at, 1311 nm and 1511 nm with an accuracy of 2×10^-3^.

Hardness was measured using a Vickers indenter (Matsuzawa) using a constant force of 100 g for a duration of 5 s. The reported value is an average of ten measurements.
